# Blood hormones and torque teno virus in peripheral blood mononuclear cells

**DOI:** 10.1016/j.heliyon.2020.e05535

**Published:** 2020-11-21

**Authors:** Peik M.A. Brundin, Britt-Marie Landgren, Peter Fjällström, Anders F. Johansson, Ivan Nalvarte

**Affiliations:** a Department of Clinical Microbiology, Infection and Immunology, The Laboratory for Molecular Infection Medicine Sweden, Umeå University, 901 87, Umeå, Sweden; b Department of Biosciences and Nutrition, Karolinska Institutet, 141 57, Huddinge, Sweden; c S:t Görans Hospital, Dept of Medicine, Unit of Infectious Diseases, 112 81, Stockholm, Sweden; d Kvinnohälsan, Karolinska University Hospital, 141 86, Huddinge, Sweden

**Keywords:** Infectious disease, Immunology, Hematology, Immune response, Immunodeficiency, Viruses, Reproductive hormone, Steroid hormones, Aging, Menstrual cycle, Estrogen, Anovulatory, Hypothyroidism, Infection, Immunity, Sex difference, Microbiome, Commensal viruses, Sex hormones

## Abstract

Men and women respond differently to infectious diseases. Women show less morbidity and mortality, partially due to the differences in sex hormone levels which can influence the immune response. Torque teno virus (TTV) is non-pathogenic and ubiquitously present in serum from a large proportion (up to 90%) of adult humans with virus levels correlating with the status of the host immune response. The source of TTV replication is unknown, but T-lymphocytes have been proposed. In this study we investigated the presence and levels of TTV in peripheral blood mononuclear cells (PBMCs) in premenopausal (pre-MP) women, post-menopausal (post-MP) women, and men, and determined their serum sex hormone levels. Of the examined subjects (*n* = 27), we found presence of TTV in PMBC from 17.6% pre-MP (*n* = 17), 25.0% post-MP (*n* = 4) and 50.0% men (*n* = 6). The levels of TTV/μg DNA were lower among TTV-positive men and post-MP women compared to pre-MP women. All the positive pre-MP women were either anovulatory, hypothyroid, or both. In addition, the TTV-positive pre-MP women had significantly lower progesterone levels compared to TTV-negative pre-MP women. Although our study was performed on a limited number of subjects, the data suggests that TTV in PBMC is associated with an anovulatory menstrual cycle with low progesterone levels, and possibly with male sex.

## 1 Introduction

Several reports indicate that females have a stronger immune response, partly due to differences in hormonal profile [1, 2]. In this paper we have investigated the role of hormones on TTV (torque teno virus), a group of commensal viruses that may be used as a secondary marker for immunity [3, 4].

There are numerous examples of animals, including humans, where females cope better than males when exposed to bacteria, virus, parasites and fungi [1, 2, 5, 6, 7, 8, 9]. In part, this may be related to the hormonal milieu, with sex hormones interacting with the immune system at multiple levels [10]. Sex hormone receptors (SHR) have been reported in various immune cells [11, 12, 13], and both the serum levels of sex hormones and the expression of SHR will determine the cellular response. The female sex hormone 17-β Estradiol (E2), the dominating form of circulating estrogen, generally acts immunostimulatory by affecting gene expression in neutrophils, macrophages, dendritic cells, CD4^+^ T-cells, CD8^+^ T-cells and B-cells, but the effect varies depending on the immune measure used [1, 14, 15]. Androgens (including testosterone and dihydrotestosterone), on the other hand, in general suppress immune cell activity with e.g. decreased expression of toll-like receptor 4 (TLR4) on macrophages, and increased expression of anti-inflammatory IL-10 [1, 16]. Thus, sex hormones (androgens, estrogens and progesterone) have distinct and overlapping effects on immune cell numbers, activity and cytokine production, which make their interaction with the immune response complex.

Incidence and severity of numerous infectious diseases show sex bias, with men having higher disease severity or pathogen load [10], and higher mortality from infectious or parasitic diseases [17]. These sex differences decline after menopause, suggesting a connection to sex hormones [17]. Sex hormone levels are also partly attributed to the risk of developing autoimmune diseases. Here, women have a higher risk of developing for example multiple sclerosis (MS), rheumatoid arthritis (RA) and systemic lupus erythematosus (SLE) [18, 19].

Consequently, differences in immune activity throughout the menstrual cycle have been reported [20]. Indeed, several autoimmune diseases (e.g. MS, RA and SLE) show fluctuations in activity during the phases of the menstrual cycle [20]. The menstrual cycle, 25–32 days long, is divided in a follicular phase, a mid-cycle ovulatory phase, and a luteal phase. Increasing and decreasing levels of 17-β estradiol and progesterone, a peak of follicle-stimulating hormone (FSH) around day 3, and a midcycle peak of luteinizing hormone (LH) characterize the different phases. Although there is limited knowledge on the impact of the menstrual cycle on immune response towards infectious diseases, it has been shown that the cytokine profile during the menstrual cycle shifts between Th1-associated and Th2-associated responses [2]. This is probably due to a biphasic effect of estrogen, where low levels of estrogen stimulate a Th1 response (cell-mediated immunity) and high levels of estrogen stimulate a Th2 response (humoral immunity) [21]. Furthermore, the number of regulatory T-cells (Treg-cells), which are important for development of autoimmunity and immune tolerance, also vary during the menstrual cycle. The number of Treg-cells are positively correlated to the serum estrogen levels [22].

Torque teno viruses (TT viruses or TTVs) are a group of highly variable single stranded DNA-viruses (family *Anelloviridae*, genus *Alphatorquetenovirus*) that consist of 29 species (TTV1-29) [3,23]. So far, there is no associated pathology to TTV infection, and it may be regarded as a commensal virus [3, 24]. Most healthy humans (up to 90%) carry several species of TTV in their blood, and the levels normally range between 2-8 log_10_ copies/mL [3, 25, 26]. Of the >3.8 × 10^10^ virions produced per day, approximately 90% are daily replaced [27, 28, 29]. Recently, TTV has received attention as a possible endogenous biomarker for immune function, with immunocompetent individuals carrying lower levels of TTV in serum than immunocompromised, indicating a suppressing role of the immune system on the viral load [3]. The high turnover-rate of virions indicates that changes in immune status can be followed in a short time frame. As with many other infectious diseases, TTV loads are also higher in men compared to women and increase with age [30].

Previous studies on TTV-levels have been performed on both plasma and PBMC, but to the best of our knowledge, none have correlated TTV-levels to hormones or the menstrual cycle [31, 32, 33]. PBMC, containing T- and B-lymphocytes, NK-cells, monocytes and a small fraction of dendritic cells, are widely used in diagnostics as sentinel markers for disease. The aim of the present study is to investigate if TTV, as a potential marker of immune function, can be detected in PBMC from healthy men and women, and whether TTV load is associated with thyroid status, sex hormone levels, and the different phases of the menstrual cycle. The differences in female and male immunity towards pathogens have implications for treatment and prevention of infectious diseases and may ultimately lead to a different approach depending on the sex of the patient.

## 2 Material and methods

### 2.1 Subjects

27 healthy individuals were included according to a protocol approved by the Central Ethical Review Board (Swedish Research Council, Stockholm, Dnr: Ö 24–2009) and consisted of 17 pre-menopausal women, 6 men and 4 postmenopausal women (Table 1). The subjects were included and sampled during 6 months (between March and September, 2010). Informed consent was obtained from the participants.

**Table 1:** Clinical information on included individuals, range (median).

	Age	Parity	Menstrual cycle length in days	Years since last menses	BMI
Pre-MP	25–37 (31)	0–2 (0)	25–31 (28)	-	17.9–27.5 (22.1)
Post-MP	58–62 (61.5)	0–4 (2)	-	6–13 (10)	21.2–34.1 (29)
Males	28–61 (51)	-	-	-	20.9–30.0 (24.5)

The inclusion criteria were: Premenopausal women aged 20–40 years with regular menstrual cycles, without hormonal contraceptives or other hormonal, anti-inflammatory (including ASA, systemic cortisone and NSAIDs) or any morphine treatment since >3 months, and parturition no later than 12 months before inclusion. Men (aged 20–70) and postmenopausal women (no menstrual bleeding since >12 months) without the above stated treatment during the last 3 months.

The exclusion criteria were: Perimenopausal women (i.e. close to menopause), medication according to the above stated criteria, and pregnancy or irregular menstrual bleedings.

### 2.2 Blood sampling and hormonal analyses

From all individuals blood was drawn at four timepoints, and for the pre-MP women Ovustick® was used to identify the LH-peak. Ovulation was then confirmed by progesterone >20 nmol/mL, 5–7 days past LH-peak. Simultaneously, at one or more timepoints PBMC was also sampled. In Pre-MP women, blood samples were drawn at the following four time-points: 1^st^ sample at Day 1–3 (early follicular phase), 2^nd^ sample at day 8–10, (mid follicular phase), 3^rd^ sample at day 12–14 (ovulatory phase) and 4^th^ 5–7 days past positive result on Ovustick® (indicating mid-luteal phase or implantation window). In post-MP women and men four samples were taken with 1-week intervals. Blood was drawn at Kvinnohälsan (Karolinska University Hospital, Huddinge) and analyzed at the Karolinska University Laboratory (KUL, Huddinge, Sweden). All samples were drawn between 8-11 a.m. PBMC fractions were prepared by centrifugation of whole blood using Vacutainer® CPT™ mononuclear cell preparation tubes (Becton Dickinson, art no. 362780) according to the manufacturer's recommendation. The buffy-coat was transferred to new tubes and slowly frozen in 20% dimethylsulphoxide (DMSO)-albumin, using isopropanol-loaded Mr. Frosty® freezing-container overnight, before long-term storage at -80 °C. Analyses were made of WBC, differential count (including B-monocytes, B-lymphocytes, B-neutrophils, B-eosinophils, B-basophils), S-TSH (thyroid stimulating hormone), S-T4, S-SHBG (sex hormone binding globulin), S-estradiol, S-testosterone, S-progesterone, S-FSH, S-LH and S-prolactin. Separate serum samples were taken and stored in -20 °C before analysis of Dihydrotestosterone (DHT) using a liquid chromatography tandem mass spectrometry (LC-MS/MS) method at Helsinki University Hospital Laboratory (HUSLAB), Helsinki, Finland. Reference values for DHT were adopted from Swerdloff *et al.* [34] and Rothman *et al.* [35].

The participants were assessed for hypo- or hyperthyroidism, and pre-MP women also whether they had a normal ovulation. A participant was considered hypothyroid if TSH >3.5 mU/L (Ref 0.4–3.5 mU/L) and anovulatory if LH was <18 nmol/L during the mid-cycle (mid follicular or ovulatory) phases and progesterone <17 nmol/L during mid luteal phase.

### 2.3 TTV DNA isolation and analysis

Frozen PBMC were gently thawed, lyzed and filter-concentrated in 7250 G (4 h, 4 °C) to a volume of ca 200μL using micro concentrators (Amicon® Ultra 2mL Ultracel®-100K, Merck Millipore, Ireland). This was performed according to QIAamp® DNA Mini and Blood Mini Handbook (Qiagen) to increase DNA yield. DNA concentration was measured using Nano-drop. DNA yield varied between 3.25-323 ng/μL, mean 58.8 ng/μL. For TTV amplification, Argene TTV R-gene® (bioMérieux S.A., Marcy l’Etoile, France) kit (described in detail by [25]) was used on an Applied Biosystems 7500 Real-time PCR system. The thermocycler was programmed according to the TTV R-gene® protocol (95 °C, 15 min followed by 45 cycles of 95 °C, 10s and 60 °C, 40s). An internal quantification standard was included in the TTV R-gene® kit. This contained pre-prepared solutions of 5, 50, 500 and 5000 copies plasmid TTV DNA per μL, as well as a sensitivity control containing 1 copy/μL. The sample wells were run in triplicates using 10 μL of concentrated DNA solution.

The detection limit was set to 1 viral particle in the sample reagent (10μL). According to the standard curve obtained, this corresponded to CT of 37.09, 42.09 and 39.14 respectively on the included three TTV qPCR plates. A sample was considered positive if 2 of 3 triplicate samples were above the detection limit (i.e. below the CT-threshold mentioned above).

### 2.4 Statistical analyses

The average hormone levels of TSH, estradiol, LH and testosterone were calculated for each of the 17 pre-MP women. The average hormone levels were used in binomial regression to explain the variance of TTV^+^/TTV^−^. Given the difference in variance in progesterone levels, LH levels and sample sizes, Welch's *t*-tests were used to test the null hypothesis of equality among pre-MP women at the 4^th^ time point (progesterone) and 3^rd^ time point (LH).

As a logistic model with logit-link, the following was used: TTV ~ log (Mean_TSH) + log (Mean_estradiol) + log (Mean_LH) + log (Mean_testosterone). In the model TTV is a dependent variable and log mean TSH, estradiol, LH and testosterone are explanatory (independent) variables. The explanatory variables are treated as covariates. No interactions were investigated. The regression model was analyzed using R 3.6.0 and RStudio 1.2.1335. dplyr 1.0.2 was used for data processing.

## 3 Results

### 3.1 Clinical data

Clinical information on age, BMI, parity, menstrual cycle length and years since last menses for all individuals are included in Table 1.

### 3.2 TTV prevalence and TTV levels

**Figure 1:**
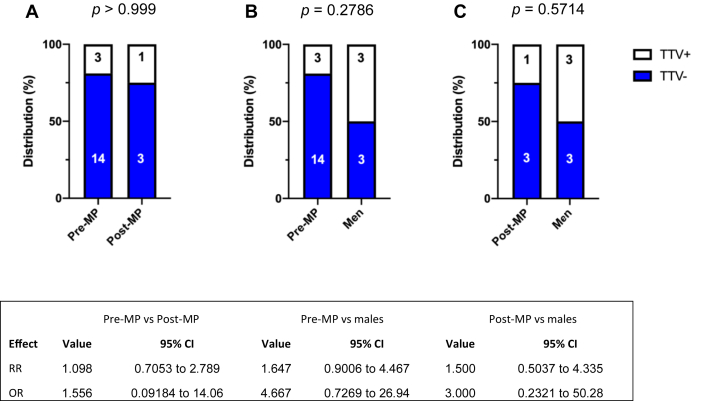
Distribution of TTV^−^ and TTV^+^ post-MP women (A) and men (B) relative to pre-MP women, and post-MP relative to men (C). Numbers in graphs indicate *n* values. Statistics tables below each graph show the respective relative risks (RR) and odds ratios (OR) following Fisher's exact test. No significance could be detected.

Of 27 included individuals (6 men, 17 pre-MP women and 4 post-MP women), in total 7 were positive for TTV in PBMC; 3 men (50.0%), 3 pre-MP women (17.6%), and 1 post-MP woman (25.0%). The detected levels of TTV were highest among the TTV positive pre-MP women and lower in the post-MP women and in the men, both in terms of detected TT viral copies/mL and when adjusting for total amount of DNA in the sample (Table 2). The differences in TTV prevalence between pre-MP and post-MP women as well as between pre-MP women and men were not statistically significant (Fisher's exact test, *p* > 0.999 and *p* = 0.2786) (Figure 1). The raw data suggested higher prevalence in men than in pre-MP women, but significance testing could not rule out a chance association (Fisher's exact test, *p* = 0.2786), possibly due to the limited number of study subjects.

**Table 2:** Clinical and hormonal data on TTV^+^ individuals, including TT virus load, thyroid status, sex hormone levels. For pre-MP women, day of the menstrual cycle, whether or not ovulation was present, range of estradiol, and peak levels of progesterone and LH, is indicated.

Subject #	12	25A^1^	25B^1^	37	24	28	31	32
Category	*Pre-MP*	*Pre-MP*	*Pre-MP*	*Pre-MP*	*Post-MP*	*Male*	*Male*	*Male*
Day of menstrual cycle	27	3	12	9	-	-	-	-
Age	29	29	29	37	58	32	53	61
BMI	21.6	33	33	27.5	27.9	20.1	25.6	30
TTV/μg DNA	9.036	4857	32.60	229.8	4.743	3.554	0.9358	3.314
Log_10_ TTV copies/mL	2.56	5.11	3,36	3.23	2.30	2.25	2.21	2.53
LH (nmol/L)	5.4	11	20	8.1	19	3.8	3.1	2.9
Max LH	8.9	21	21	14	-	-	-	-
Progesterone (nmol/L)	4	2.1	2.2	3.2	<1.0	<1.0	1	1.8
Max Progesterone	11	9.8	9.8	20	-	-	-	-
Testosterone (nmol/L)	1.2	1.5	1.8	0.5	<0.4	18	13	11
DHT (nmol/L)	0.2	0.2	0.5	0.5	0	1.3	1.8	1.2
Estradiol (pmol/L)	353	164	<150	392	27	46	105	36
Range Estradiol	189–353	<150–301	<150–301	<150–1030	-	-	-	-
FSH (U/L)	3.6	5.5	6	23	52	3.6	2.8	5.6
Range FSH	2–5.1	4.1–6	4.1–6	5.8–23	-	-	-	-
Thyroid status^2^	*Euthyroid*	*Hypothyroid*	*Hypothyroid*	*Hypothyroid*	*Euthyroid*	*Euthyroid*	*Euthyroid*	*Euthyroid*
Ovulation^3^	*No*	*No*	*No*	*Yes*	-	-	-	-

Abbreviations: Luteinizing hormone (LH). Dihydrotestosterone (DHT). Follicle-stimulating hormone (FSH).

Reference values:

Pre-MP females: S-17β-estradiol (follicular phase) 100–200 pmol/L; (ovulatory phase) 500–1500; (luteal phase) 200–800. S-FSH (follicular phase) 2.5–10 U/L; (ovulatory phase) 4.0–14; (luteal phase) 0.7–8.5. S-LH (follicular phase) 1.8–12 nmol/L, (ovulatory phase) 18–90, (luteal phase) 0.6–15. S-progesterone, (follicular phase) < 4.8 nmol/L; (luteal phase) > 17. S-Testosterone <2.7 nmol/L. DHT ~0.3 nmol/L.

Post-MP females: S-17β-estradiol <50 pmol/L; S-FSH 25–150 U/L; S-LH 18–78 nmol/L; S-Progesterone <3.0 nmol/L; S-Testosterone <2.7 nmol/L. DHT ~0.1 nmol/L.

Males: S-17β-estradiol 50–150 pmol/L; S-FSH: 1.0–12.5 U/L; S-LH 1.2–9.6 nmol/L; S-Progesterone <3.0 nmol/L; S-Testosterone 10–30 nmol/L. DHT 0.38–3.27 nmol/L.

1 25A and 25B represents samples of one individual at two different timepoints.

2 Hypothyroidism is defined as S-thyroid-stimulating hormone (TSH) > 3.5 mU/L.

3 Anovulation is defined as LH < 18 nmol/L in ovulatory phase and progesterone <17 nmol/L in the luteal phase.

### 3.3 Hormonal status in TTV-positive pre-MP women

To determine whether sex hormones influence the risk of being TTV-positive (TTV^+^) we compared the average sex hormone levels in TTV^+^ (*n* = 3) and TTV^−^ (*n* = 14) individuals using a binomial regression including S-estradiol, S-testosterone, S-LH and S-TSH. The results showed no significant relationship between hormone levels and TTV-status (Table 3).

**Table 3:** Binominal regression analysis of thyroid stimulating hormone (TSH), prolactin, and luteinizing hormone (LH). Dispersion parameter for binominal family taken to be 1. Null deviance: 1.6220 × 10^1^ on 17 degrees of freedom (Df). Residual deviance: 5.5798 on 13 Df. Akaike information criterion (AIC): 15.58. Number of Fisher Scoring iterations: 10.

Deviance residuals				
Min	1Q	Median	3Q	Max
-1.356	-0.1560	-0.00543	-0.00032	-1.622
*Coefficients:*	*Estimate*	*Standard error*	*Z value*	*Pr (>|z|)*
Intercept	-6.611	29.67	-0.223	0.824
Log Average TSH	16.291	16.97	0.960	0.337
Log Average Estradiol	0.1415	5.239	0.027	0.978
Log Average LH	-2.651	3.599	-0.737	0.461
Log Average Testosterone	-7.593	14.05	-0.541	0.589

We noted that out of three TTV^+^ pre-MP women, two (# 12 and 25) were aberrant in their hormonal status and did not ovulate. Two (#25 and 37) also had laboratory signs of hypothyroidism, of which one (#25) had an exceptionally high viral load (Table 2).

**Figure 2:**
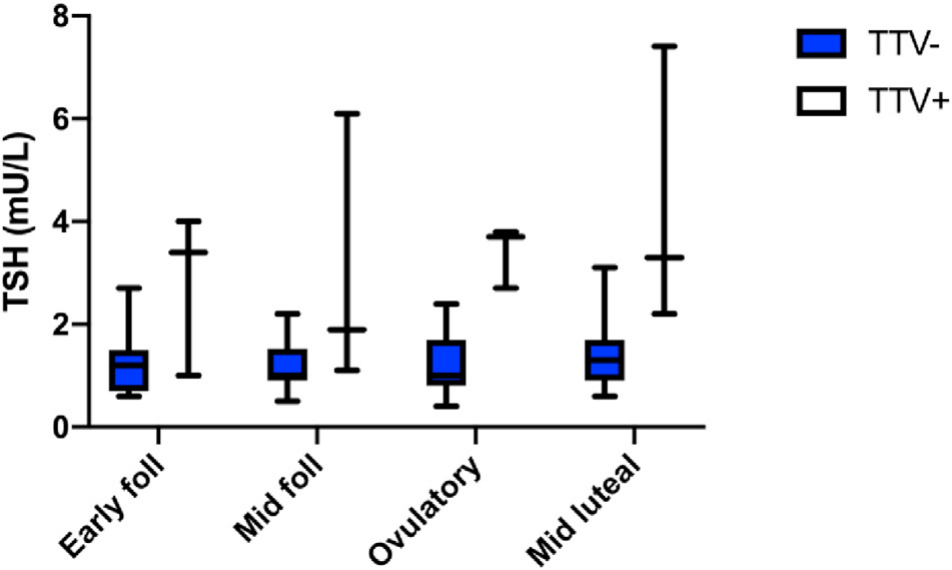
TSH-levels in TTV^−^ (Neg, *n* = 14) and TTV^+^ (Pos, *n* = 3) pre-MP women, divided by phase of the menstrual cycle. Of the three TTV^+^, one was anovulatory, one hypothyroid with normal ovulation and one both anovulatory and hypothyroid. None of the TTV^−^ were hypothyroid. Hypothyroidism was defined by TSH >3.5 mU/L at one or more occasions.

None of the TTV^−^ pre-MP women had signs of hypothyroidism, i.e. normal TSH-levels (range 0.4–3.2, average 1.27 mU/L, Ref 0.4–3.5 mU/L). The boxplot of TSH comparing TTV^+^ and TTV^−^-individuals (Figure 2) indicates a distinction between the two groups. However, when comparing average TSH from TTV^+^ and TTV^−^ individuals in a binominal regression, there was no significant difference (*p*-value = 0.337, Table 3). To establish if this result could be due to a power problem, a bootstrap power analysis was performed and showed that a binomial regression with given group sizes; standard deviation and average difference in TSH-levels, had 11.7% probability only to detect this mean difference. Additional binominal regression analysis on serum hormones showed that it was not meaningful to further analyze average values.

**Figure 3:**
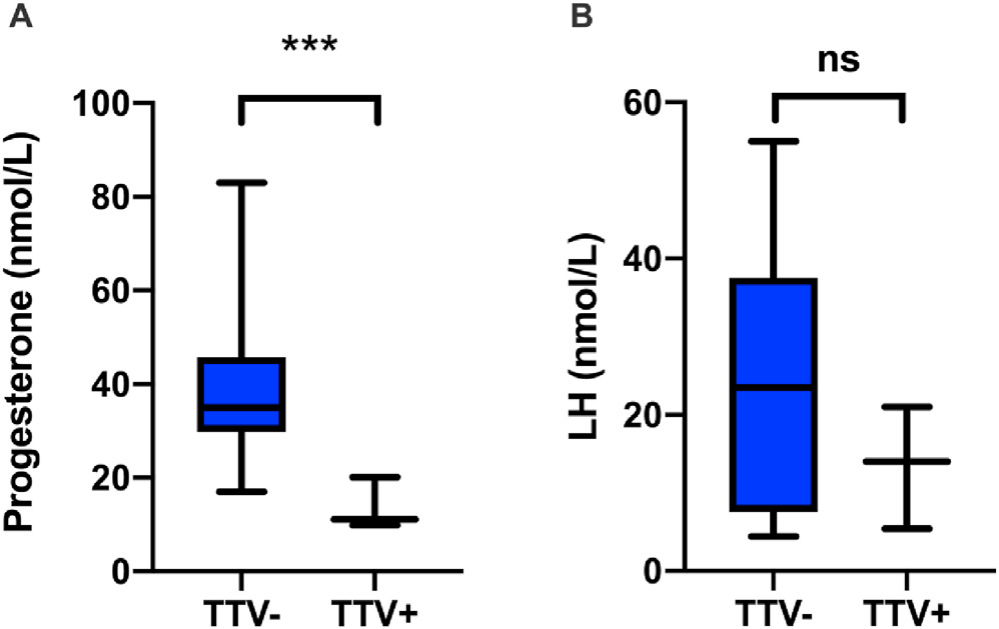
Levels of progesterone (mid-luteal phase) and LH (ovulatory phase) in TTV^+^ (*n* = 3) and TTV^−^ (*n* = 14) pre-MP women. Standard deviation is shown in error bars. The average S-progesterone levels (A) were significantly different in a two-tailed Student's *t*-test, using Welch's correction (*p* = 0.002, *t* = 3.989, Df = 12.53). Difference in average S-LH were not statistically significant (*p* = 0,156, *t* = 1.5937, Df = 6.874).

The levels of progesterone and LH during the menstrual cycle are important indicators for ovulation. Progesterone levels are normally expected to rise during mid-luteal phase. Samples from the mid-luteal phase revealed (using Welch's t-test) significantly lower progesterone levels (p = 0.002) in TTV^+^ compared to TTV^−^ pre-MP women (Figure 3A). Notably, three out of four TTV^+^ samples were from the early phases of the menstrual cycle, when progesterone is also low (Table 2). LH that is expected to peak at ovulatory phase was low, but not significantly different in TTV^+^ as compared to TTV^−^ (Figure 3B). Low LH together with low progesterone indicates anovulation.

Altogether, all 3 TTV^+^ pre-MP women showed hormonal aberrance. One was anovulatory, one hypothyroid with normal ovulation, and one both hypothyroid and anovulatory (Table 2). None of the TTV^−^ individuals had signs of anovulation or irregularities in thyroid hormone levels. Although this result hindered us from determining if menstrual cycle phases could be linked to TTV load in PBMCs, we noted that of 4 TTV^+^ samples, 3 were obtained from the follicular phase (day 3, 9 and 12) and 1 from the luteal phase (day 27) (Table 2 and Table 4A).

**Table 4:** Amount of TTV (TTV particles/μg template DNA) in investigated subjects using qPCR. TTV status, (+) or (-), was determined according to whether amplification of TTV-DNA was present in the respective samples and whether the qPCR was above cut-off (1.0 viral particle per sample, i.e. qPCR reaction well (10 μL)). 4A. Pre-MP women (sampled four times through the menstrual cycle for TTV). 4B Post-MP women and men (sampled once for TTV). *Not detected* indicates no detection signal on the qPCR assay.

4A							
	Subject #	Early follicular	Mid follicular	Ovulatory	Mid luteal	TTV status	Comment
Pre-MP (sampled 4 times)	4	N A	N A	Not detected	Not detected	-	Not detected
	12	B C	B C	*9.036*	*B C*	+	+
	13	Not detected	Not detected	Not detected	Not detected	-	Not detected
	15	N A	Not detected	Not detected	Not detected	-	Not detected
	18	N A	N A	Not detected	Not detected	-	Not detected
	21	Not detected	Not detected	Not detected	Not detected	-	Not detected
	22	N A	Not detected	Not detected	Not detected	-	Not detected
	25	*4857*	*32.60*	B C	N A	+	+
	30	Not detected	Not detected	Not detected	Not detected	-	Not detected
	35	Not detected	Not detected	Not detected	Not detected	-	Not detected
	36	Not detected	Not detected	Not detected	Not detected	-	Not detected
	37	B C	*229.8*	B C	N A	+	+
	38	B C	N A	N A	N A	-	B C
	39	N A	Not detected	B C	N A	-	B C
	41	N A	Not detected	Not detected	Not detected	-	Not detected
	42	N A	Not detected	Not detected	Not detected	-	Not detected
	43	B C	B C	B C	B C	-	B C
4B							
	**Subject #**					**TTV status**	**Comment**
Post-MP (Sampled once)	3	B C				-	B C
	14	Not detected				-	Not detected
	16	B C				-	B C
	24	*4.743*				+	+
Men (Sampled once)	23	B C				*-*	B C
	27	B C				*-*	B C
	28	*3.554*				+	+
	31	*0.9358*				+	+
	32	*3.314*				+	+
	40	B C				-	B C

Abbreviations: Not available (N A). Below cut-off (B C).

### 3.4 Hormonal status in men and post-MP women

All three TTV^−^ men, and two out of three TTV^−^ post-MP women hade traces of TTV, but below cut-off (Table 4B). All men and post-MP women (TTV^+^ and TTV^−^) had normal (euthyroid) levels of TSH. Due to the low number of participants, it was not possible to use regression models for analyzing hormone levels in relation to TTV status in men and post-MP women. TTV status, age and range of hormone levels are shown in Table 5.

**Table 5:** TTV-status, age and range of Thyroid-stimulating hormone (TSH), estradiol (E2), luteinizing hormone (LH) and testosterone in postmenopausal women and men.

	Subject #	Range TSH	TTV +/-	TTV comment	Range E2	Range LH	Range testosterone	Age
Postmenopausal	3	1.9–2.2	-	B C	19–35	42–48	1.4–1.9	62
women	14	1.3–1.4	-	N A	47–59	22–27	0.6–1.0	63
	16	0.6–1.7	-	B C	31–53	22–27	0.8–1.1	61
	24	1.6–2.2	+	positive	27–46	19–23	<0.4–0.6	58
Men	23	1.1–1,8	-	B C	53–66	3–4.2	13–17	49
	27	2.2–2.7	-	B C	74–125	3.6–4.7	11.20	28
	28	1.3–2.2	+	positive	45–52	3.8–4.8	15–18	32
	31	1.0–1.4	+	positive	40–105	2.8–5.5	10.13	53
	32	1.7–2.0	+	positive	36–50	2.5–3.4	10.11	61
	40	2.4–3.2	-	B C	43–61	3.3–5.1	10.12	55

Abbreviations: Not determined (N A). Below cut-off (B C).

## 4 Discussion

The detection of TTV in 17.6% pre-MP females, 25.0% post-MP females, and 50.0% males suggests that TTV presence in the PBMC fraction of peripheral blood may be associated with sex. This observation supports findings from previous studies [30]. Our observations also suggest that TTV presence in the PBMC fraction of peripheral blood is related to hormone levels.

TTV^+^ samples from pre-MP women in our cohort were mostly found during the first half of the menstrual cycle, and pre-MP women who were positive for TTV had hormonal aberrances being either anovulatory, hypothyroid or both. As expected, when being anovulatory, the TTV^+^ pre-MP women differ in levels of S-LH and S-progesterone compared to the TTV^−^ women (Figure 3). The TTV levels in PBMC were in the lower range (2.53–5.11 log_10_ copies/mL, median 2.55) compared to the levels previously reported in plasma (2–8 log_10_ copies/mL) [3, 25] (Table 2). As stated above, all included men (*n* = 6) and post-MP women (*n* = 4) were euthyroid. The small number of men and post-MP women included in this study did not permit us to draw any conclusions whether TTV status in these groups correlate to sex hormone levels. Undeniably, the low number of participants included, set constraints on the generalization of our findings.

Sex differences in immunity are affected by several factors, including social, behavioral and genetic (related to differences in sex chromosomes) [2, 5, 19, 36]. Social and behavioral factors may certainly contribute to sex differences in mortality from infectious diseases, however, even when controlling for exposure to infection in animal models, females seems to mount a stronger response towards pathogens [37].

Interactions of sex hormones with the immune system are established on multiple levels (reviewed in e.g. [10, 15, 16]). Estrogen and testosterone often have opposite effects; estrogen acting pro-inflammatory, testosterone anti-inflammatory. An interesting finding is that estrogen seems to stimulate anti-apoptotic properties in B-cells and increase somatic hypermutations and the class switch of immunoglobulins [38]. These factors have been proposed as plausible reasons to why both an increased protective effect against pathogens and an increased risk of autoimmunity may be observed in pre-MP women [38]. Estrogen promotes the cell-mediated response in low concentrations, and the humoral response in high concentrations [15]. Yet, we did not see any association of TTV load to estrogen levels in the present study. Progesterone affects both the innate immune response (e.g. by suppressing activation of macrophages and dendritic cells, inhibiting production of proinflammatory cytokines and stimulating anti-inflammatory cytokines) [39], and the adaptive immunity (decreased class switch of immunoglobulins and somatic hypermutations, and decreased ability to present antigens in B-cells) [40, 41]. Previous reports state that a low progesterone level is protective against pathogens, and higher levels increase the risk of infections (e.g. herpes, HIV and tuberculosis), but examples of the opposite exists (e.g. influenza, *Salmonella typhimurium* and *Clostridioides difficile*) [6, 39, 42]. In our study, low progesterone was correlated to TTV^+^ in pre-MP women.

T-lymphocytes constitute the major cell type of PBMCs and have been pointed out as a possible source of TTV replication [43, 44]. Yet, either low levels or no TTV could be detected in our PBMC material. Based on our findings, we suggest that TTV do not replicate in non-activated PBMC. However, it is possible that one or more of the smaller cell subsets of PBMC contain replicating TTV. In this study, no difference in absolute lymphocyte count (ALC) could be seen between TTV^+^ and TTV^−^ pre-MP individuals (data not shown). More studies on larger cohorts are needed to evaluate differences in TTV-replication in men and women.

Using plasma, Haloschan *et al.* showed that TTV is generally found in lower levels in women than men of the same age [30]. Further, they reported in general lower TTV levels in pre-MP women than in men and post-MP women. This corresponds well with the hypothesis that men and women have a difference in immune response to infection related to sex hormones [1].

In a study by Maggi *et al.* (2001), TTV-levels were considerably lower in PBMC than in plasma from the same individuals [31]. Fernández-Ruíz *et al.* estimated TTV-levels in 221 kidney transplant recipients, and found that TTV-levels were higher among patients that subsequently developed post-transplant infection or immune related adverse events (iRAE) [45]. To compare the results (on plasma) from Fernández-Ruíz and colleagues with our findings (on PBMC), we decided to use the same method (PCR amplification kit from Argene, TTV R-gene®). Our results indicate TTV-levels in the same range in PBMC as in their base-line samples of plasma (i.e. before kidney transplantation and immunosuppression). However, only 2.4% of the plasma samples in the study from Fernández-Ruíz *et al.* [45], were below lower limit of detection, whereas in our study on PBMC, the majority of individuals (74.1%) were below cut-off.

The results from the present study add information on hormone levels in blood to previous investigations of TTV in PBMC (e.g. [31, 46, 47]). We suggest that our results, with TTV not detected in PBMC from healthy (ovulating and euthyroid) pre-MP women, reflect the lower viral load found in plasma from pre-MP women compared to plasma from men and post-MP women (as reported in [31]). The comparably higher TTV load that we do find in TTV + pre-MP women, are only found in hormonally aberrant individuals. Therefore, we hypothesize that the hormonal deviation modifies the immune response, which would make the host more vulnerable to TTV replication.

Using TTV-levels to assess immune status is appealing, as it has been proposed to reflect the *functional* immune response of an individual [3]. This could prove to be a better option than measuring single lab parameters as absolute neutrophil count (ANC), absolute CD4^+^ cell counts, or (for patients on immunosuppressant treatment) pharmacological concentrations of immunosuppressant drugs. Drug concentrations depend on individual variation in pharmacodynamics (PD), and therefore may not reflect the true concentration in patient tissues. Also, sex differences in PD implies that the same serum concentration does not necessarily lead to the same effect in men and women [2].

One of few studies on pathogen immunity and viral load related to the menstrual cycle was performed by Benki *et al.* [48]. Their results showed that viral shedding of HIV in cervical secretions varied during the menstrual cycle, and the lowest levels of virus in cervix were obtained during the days closest to the LH-peak in serum. In this study we observed that in pre-MP women, progesterone is significantly lower in TTV^+^ subjects. A low LH-surge means that ovulation will not take place. Normally, the ovum will form a *corpus luteum* which will continue to produce progesterone, creating the expected increase of progesterone in the luteal phase of the menstrual cycle. If ovulation does not occur, the rise of progesterone will be absent. TTV^+^ individuals were related to *low* progesterone in the present study, but most literature states that *high* progesterone levels increase the risk of infections. Previous studies have shown that both LH, TSH and thyroid hormones (TH) have immuno-modulating properties [49, 50, 51]. Ovulation with prior rise of LH may be described as an inflammatory process important for a functional menstrual cycle [52]. We suspect that the absent LH-surge (and possibly high TSH with following absence of TH through negative feedback) may be involved in modulation of the immune system rather than progesterone.

In line with the mentioned reports ([49, 50, 51]) and the findings from Benki *et al.* [48] we suggest that anovulation and the absence of a normal LH-peak in our data contributes to a favorable environment for TTV replication.

In summary, our findings suggest that TTV in PBMC is associated with an anovulatory menstrual cycle (with low increase of serum LH and progesterone), and possibly associated with male sex. However, these results have to be considered in the light of the low number of participants, which limits generalization of the findings.

Furthermore, two out of 3 TTV^+^ pre-MP women had signs of hypothyroidism with elevated TSH. Preferably, future studies including sex and hormonal status (e.g. pre- or post-menopause, contraceptives, hormonal replacement therapies and ovulation, as well as signs of hypothyroidism), should be performed to obtain more information on the impact of the menstrual cycle on TTV load and immune response.

## Declarations

### Author contribution statement

P. Brundin: Conceived and designed the experiments; Performed the experiments; Analyzed and interpreted the data; Wrote the paper.

B-M. Landgren, P. Fjällström and A. Johansson: Analyzed and interpreted the data.

I. Nalvarte: Conceived and designed the experiments; Analyzed and interpreted the data; Contributed reagents, materials, analysis tools or data.

### Funding statement

This work was supported by the 10.13039/501100004047Karolinska Institutet (FS-2018:0007), Region Västerbotten (RV-866221) and Folksams Forskningsstiftelse.

### Declaration of interests statement

The authors declare no conflict of interest.

### Additional information

No additional information is available for this paper.

## References

[1] Klein S.L., Flanagan K.L. (2016). Sex differences in immune responses. Nat. Rev. Immunol..

[2] Fish E.N. (2008). The X-files in immunity: sex-based differences predispose immune responses. Nat. Rev. Immunol..

[3] Focosi D., Antonelli G., Pistello M., Maggi F. (2016). Torquetenovirus: the human virome from bench to bedside. Clin. Microbiol. Infect..

[4] Rezahosseini O., Drabe C.H., Sorensen S.S. (2019). Torque-Teno virus viral load as a potential endogenous marker of immune function in solid organ transplantation. Transplant. Rev..

[5] vom Steeg L.G., Klein S.L. (2016). SeXX matters in infectious disease pathogenesis. PLoS Pathog..

[6] Vazquez-Martinez E.R., Garcia-Gomez E., Camacho-Arroyo I., Gonzalez-Pedrajo B. (2018). Sexual dimorphism in bacterial infections. Biol. Sex Differ..

[7] Guerra-Silveira F., Abad-Franch F. (2013). Sex bias in infectious disease epidemiology: patterns and processes. PloS One.

[8] Restrepo A., Salazar M.E., Cano L.E., Stover E.P., Feldman D., Stevens D.A. (1984). Estrogens inhibit mycelium-to-yeast transformation in the fungus Paracoccidioides brasiliensis: implications for resistance of females to paracoccidioidomycosis. Infect. Immun..

[9] Hamid Salim M.A., Declercq E., Van Deun A., Saki K.A. (2004). Gender differences in tuberculosis: a prevalence survey done in Bangladesh. Int. J. Tubercul. Lung Dis..

[10] Klein S.L., Roberts C.W. (2010). Sex Hormones and Immunity to Infection.

[11] Danel L., Souweine G., Monier J.C., Saez S. (1983). Specific estrogen binding sites in human lymphoid cells and thymic cells. J. Steroid Biochem..

[12] Cutolo M., Accardo S., Villaggio B. (1996). Androgen and estrogen receptors are present in primary cultures of human synovial macrophages. J. Clin. Endocrinol. Metab..

[13] Phiel K.L., Henderson R.A., Adelman S.J., Elloso M.M. (2005). Differential estrogen receptor gene expression in human peripheral blood mononuclear cell populations. Immunol. Lett..

[14] Khan D., Ansar Ahmed S. (2015). The immune system is a natural target for estrogen action: opposing effects of estrogen in two prototypical autoimmune diseases. Front. Immunol..

[15] Straub R.H. (2007). The complex role of estrogens in inflammation. Endocr. Rev..

[16] Foo Y.Z., Nakagawa S., Rhodes G., Simmons L.W. (2017). The effects of sex hormones on immune function: a meta-analysis. Biol. Rev. Camb. Phil. Soc..

[17] Owens I.P. (2002). Ecology and evolution. Sex differences in mortality rate. Science.

[18] Whitacre C.C. (2001). Sex differences in autoimmune disease. Nat. Immunol..

[19] Pennell L.M., Galligan C.L., Fish E.N. (2012). Sex affects immunity. J. Autoimmun..

[20] Oertelt-Prigione S. (2012). Immunology and the menstrual cycle. Autoimmun. Rev..

[21] Gilmore W., Weiner L.P., Correale J. (1997). Effect of estradiol on cytokine secretion by proteolipid protein-specific T cell clones isolated from multiple sclerosis patients and normal control subjects. J. Immunol..

[22] Arruvito L., Sanz M., Banham A.H., Fainboim L. (2007). Expansion of CD4+CD25+and FOXP3+ regulatory T cells during the follicular phase of the menstrual cycle: implications for human reproduction. J. Immunol..

[23] King A.M.Q., Adams M.J., Carstens E.B., Lefkowitz E.J. (2012). Anelloviridae. Virus Taxonomy.

[24] Griffiths P. (1999). Time to consider the concept of a commensal virus?. Rev. Med. Virol..

[25] Kulifaj D., Durgueil-Lariviere B., Meynier F. (2018). Development of a standardized real time PCR for Torque teno viruses (TTV) viral load detection and quantification: a new tool for immune monitoring. J. Clin. Virol..

[26] Spandole S., Cimponeriu D., Berca L.M., Mihaescu G. (2015). Human anelloviruses: an update of molecular, epidemiological and clinical aspects. Arch. Virol..

[27] Ball J.K., Curran R., Berridge S. (1999). TT virus sequence heterogeneity in vivo: evidence for co-infection with multiple genetic types. J. Gen. Virol..

[28] Maggi F., Bendinelli M. (2010). Human anelloviruses and the central nervous system. Rev. Med. Virol..

[29] Maggi F., Bendinelli M., de Villiers Ethel-Michele (2009). Immunobiology of the torque teno viruses and other anelloviruses. TT Viruses the Still Elusive Human Pathogens.

[30] Haloschan M., Bettesch R., Gorzer I., Weseslindtner L., Kundi M., Puchhammer-Stockl E. (2014). TTV DNA plasma load and its association with age, gender, and HCMV IgG serostatus in healthy adults. Age.

[31] Maggi F., Fornai C., Zaccaro L. (2001). TT virus (TTV) loads associated with different peripheral blood cell types and evidence for TTV replication in activated mononuclear cells. J. Med. Virol..

[32] Maggi F., Focosi D., Albani M. (2010). Role of hematopoietic cells in the maintenance of chronic human torquetenovirus plasma viremia. J. Virol..

[33] Maggi F., Tempestini E., Lanini L. (2005). Blood levels of TT virus following immune stimulation with influenza or hepatitis B vaccine. J. Med. Virol..

[34] Swerdloff R.S., Dudley R.E., Page S.T., Wang C., Salameh W.A. (2017). Dihydrotestosterone: biochemistry, physiology, and clinical implications of elevated blood levels. Endocr. Rev..

[35] Rothman M.S., Carlson N.E., Xu M. (2011). Reexamination of testosterone, dihydrotestosterone, estradiol and estrone levels across the menstrual cycle and in postmenopausal women measured by liquid chromatography-tandem mass spectrometry. Steroids.

[36] Schurz H., Salie M., Tromp G., Hoal E.G., Kinnear C.J., Moller M. (2019). The X chromosome and sex-specific effects in infectious disease susceptibility. Hum. Genom..

[37] Daniels C.W., Belosevic M. (1994). Serum antibody responses by male and female C57Bl/6 mice infected with Giardia muris. Clin. Exp. Immunol..

[38] Giefing-Kroll C., Berger P., Lepperdinger G., Grubeck-Loebenstein B. (2015). How sex and age affect immune responses, susceptibility to infections, and response to vaccination. Aging Cell.

[39] Hall O.J., Klein S.L. (2017). Progesterone-based compounds affect immune responses and susceptibility to infections at diverse mucosal sites. Mucosal Immunol..

[40] Sakiani S., Olsen N.J., Kovacs W.J. (2013). Gonadal steroids and humoral immunity. Nat. Rev. Endocrinol..

[41] Zhang L., Chang K.K., Li M.Q., Li D.J., Yao X.Y. (2013 Dec 15). Mouse endometrial stromal cells and progesterone inhibit the activation and regulate the differentiation and antibody secretion of mouse B cells. Int. J. Clin. Exp. Pathol..

[42] Kaushic C., Ashkar A.A., Reid L.A., Rosenthal K.L. (2003). Progesterone increases susceptibility and decreases immune responses to genital herpes infection. J. Virol..

[43] Maggi F., Ricci V., Bendinelli M. (2008). Changes in CD8+57+ T lymphocyte expansions after autologous hematopoietic stem cell transplantation correlate with changes in torquetenovirus viremia. Transplantation.

[44] Focosi D., Macera L., Boggi U., Nelli L.C., Maggi F. (2015). Short-term kinetics of torque teno virus viraemia after induction immunosuppression confirm T lymphocytes as the main replication-competent cells. J. Gen. Virol..

[45] Fernandez-Ruiz M., Albert E., Gimenez E. (2019). Monitoring of alphatorquevirus DNA levels for the prediction of immunosuppression-related complications after kidney transplantation. Am. J. Transplant..

[46] Mariscal L.F., Lopez-Alcorocho J.M., Rodriguez-Inigo E. (2002). TT virus replicates in stimulated but not in nonstimulated peripheral blood mononuclear cells. Virology.

[47] Okamura A., Yoshioka M., Kubota M., Kikuta H., Ishiko H., Kobayashi K. (1999). Detection of a novel DNA virus (TTV) sequence in peripheral blood mononuclear cells. J. Med. Virol..

[48] Benki S., Mostad S.B., Richardson B.A., Mandaliya K., Kreiss J.K., Overbaugh J. (2004). Cyclic shedding of HIV-1 RNA in cervical secretions during the menstrual cycle. J. Infect. Dis..

[49] Berczi I. (1997). Pituitary hormones and immune function. Acta Paediatr. Suppl..

[50] Berczi I., Chalmers I.M., Nagy E., Warrington R.J. (1996). The immune effects of neuropeptides. Bailliere. Clin. Rheumatol..

[51] Jara E.L., Munoz-Durango N., Llanos C. (2017). Modulating the function of the immune system by thyroid hormones and thyrotropin. Immunol. Lett..

[52] Duffy D.M., Ko C., Jo M., Brannstrom M., Curry T.E. (2019). Ovulation: parallels with inflammatory processes. Endocr. Rev..

